# A Small Molecule Inhibitor of Human RAD51 Potentiates Breast Cancer Cell Killing by Therapeutic Agents in Mouse Xenografts

**DOI:** 10.1371/journal.pone.0100993

**Published:** 2014-06-27

**Authors:** Fei Huang, Alexander V. Mazin

**Affiliations:** Department of Biochemistry and Molecular Biology, Drexel University College of Medicine, Philadelphia, Pennsylvania, United States of America; Saint Louis University, United States of America

## Abstract

The homologous recombination pathway is responsible for the repair of DNA double strand breaks. RAD51, a key homologous recombination protein, promotes the search for homology and DNA strand exchange between homologous DNA molecules. RAD51 is overexpressed in a variety of cancer cells. Downregulation of RAD51 by siRNA increases radio- or chemo-sensitivity of cancer cells. We recently developed a specific RAD51 small molecule inhibitor, B02, which inhibits DNA strand exchange activity of RAD51 *in* *vitro*. In this study, we used human breast cancer cells MDA-MB-231 to investigate the ability of B02 to inhibit RAD51 and to potentiate an anti-cancer effect of chemotherapeutic agents including doxorubicin, etoposide, topotecan, and cisplatin. We found that the combination of B02 with cisplatin has the strongest killing effect on the cancer cells. We then tested the effect of B02 and cisplatin on the MDA-MB-231 cell proliferation in mouse xenografts. Our results showed that B02 significantly enhances the therapeutic effect of cisplatin on tumor cells *in* *vivo*. Our current data demonstrate that use of RAD51-specific small molecule inhibitor represents a feasible strategy of a combination anti-cancer therapy.

## Introduction

Among DNA repair pathways, homologous recombination (HR) is especially important for the repair DNA double-strand breaks (DSBs) and inter-strand crosslinks (ICLs), most harmful types of DNA damage [1]. RAD51 protein plays a central role in the HR pathway [2]. It forms a helical filament on single-strand DNA (ssDNA) overhangs, which are generated exonucleolitycally on DSBs sites at presynaptic stage [3]. The filament performs the search for homologous double-strand DNA (dsDNA) and carries out an invasion of ssDNA into homologous dsDNA that is used as a template by DNA polymerase to retrieve genetic information required for DSB repair [4]. In addition to its direct role in DSB repair, yeast Rad51 was reported to play an important role during replication of DNA damaged by alkylating agent MMS [5]. In response to DNA damage, RAD51 with several other DNA repair proteins was observed to form nuclear foci, which are thought to represent cellular structures responsible for DNA repair [6].

Because of intrinsic genome instability many cancer cells become deficient in different DNA repair pathways [7], [8], [9]. In turn, DNA repair deficiency may further foster genome instability of cancer cells generating a positive feedback loop that accelerates malignant transformation [10]. Loss of some of the DNA repair pathways may increase dependence of cancer cells survival on the remaining pathways, among which HR seems to be especially important for cancer cell survival. **Thus**, overexperssion of RAD51, a central HR protein, was observed in a variety of tumor cells and represents a hallmark of cancer which has been used as a diagnostic marker of certain cancers [11], [12], [13], [14]. It is thought that RAD51 overexpression may provide a mechanism to compensate for the deficiency in alternative DNA repair pathways helping cancer cells to survive [15]. Similar to RAD51, several other HR proteins including XRCC3, RAD52 and RAD54 show a 2 to 5-fold increase in mRNA and protein levels in cancer cells indicating an important role of the entire HR pathway in tumorigenesis [16]. In contrast, no significant differences were observed in cancer and normal cells in expression of the genes relating to the non-homologous end-joining (NHEJ) pathway, an alternative pathway of DSBs repair which operates through a template-independent error-prone mechanism [16].

It was reported that RAD51 overexpression contributes to chemoresistance in human soft tissue sarcoma cells [17] and rescues radiation sensitivity of BRCA2-defective cancer cells [18]. Given strong correlation between RAD51 overexpression and cancer, RAD51 has been recognized as an important target for novel anti-cancer therapies. Because cells cannot circumvent even one unrepaired DSB, inactivation of RAD51 is expected to increase cell sensitivity to DNA damage [19]. Indeed, down-regulation of RAD51 by siRNA was successfully used to sensitize HeLa cells to cisplatin [20], [21].

Recently, by high throughput screening we identified a specific RAD51 small molecule inhibitor (*E*)-3-benzyl-2-(2-(pyridin-3-yl) vinyl) quinazolin-4(3H)-one, named B02 [22]. We showed that B02 efficiently and specifically inhibited DNA strand exchange activity of RAD51. Importantly, B02 showed biological effect in human and mouse cells. Using human embryonic kidney (HEK) cells we showed that B02 disrupted RAD51 foci formation in response to DNA damage and inhibited DSB repair and DSB-dependent HR [23].

Here, we wished to test whether B02 can increase the sensitivity of cancer cells to chemotherapeutic DNA damaging agents. A high expression level of RAD51 observed in cancer cells could potentially put a stronger demand on B02 concentration to achieve cell sensitization to chemotherapeutic agents. Using breast cancer MDA-MB-231 cells, we tested the effect of B02 on cell killing by several clinically approved anticancer drugs, such as doxorubicin, etoposide, topotecan and cisplatin. MDA-MB-231 is a triple negative breast cancer (TNBC) (estrogen-receptor, progesterone-receptor and *HER2* gene amplification negative) cell line, which is highly aggressive. Patients with TNBC have as much as a two-fold higher probability than other breast cancer patients to develop metastasis and suffer shorter survival [24]. We observed an enhancement in MDA-MB-231 cell killing by B02 with all tested chemotherapeutic drugs, with the strongest effect observed for B02 combination with cisplatin. Using mouse xenografts, we then tested the therapeutic effect of B02 in combination with cisplatin on MDA-MB-231 cells *in* *vivo.* Our results demonstrated that B02 significantly sensitizes MDA-MB-231 breast cancer cells to cisplatin *in* *vivo*. We also found that B02 efficiently disrupts RAD51 foci formation in MDA-MB-231 cells in mouse xenografts indicating that B02 acts by targeting RAD51 protein *in* *vivo.* Thus, our current data demonstrate that use of RAD51-specific small molecule inhibitor represents a feasible strategy of a combination anti-cancer therapy.

## Materials and Methods

### Cell culture

MDA-MB-231-luc cell line was obtained from Cell Biolabs, and MDA-MB-231 was a gift of Dr. Reginato. Cells were cultured in complete DMEM media composed of DMEM (Sigma; D6429) supplemented with 10% fetal bovine serum (Gibco), 100 units ml^−1^ penicillin and 100 µg ml^−1^ streptomycin in a humidified atmosphere containing 5% CO_2_ at 37°C.

### Animals

This study was carried out in strict accordance with the recommendations in the Guide for the Care and Use of Laboratory Animals of the National Institutes of Health and was approved by the Institutional Animal Care and Use Committee (IACUC) of Drexel University College of Medicine (IACUC Animal Use Protocol #20030). All surgery was performed under isoflurane anesthesia, and all efforts were made to minimize suffering. Female, athymic, NCR nude mice (Taconic Farms, Hudson, NY) of 8 weeks and weighing 18–20 g were used in experiments. The welfare of mice was assessed by the weight and body condition scoring (BCS) as described [25].

### Chemicals

B02 was purchased from Ryan Scientific and Seven Hills Chemical Inc. Cisplatin was purchased from Sigma and was reconstituted in normal saline (NS) containing 0.9% NaCl. In experiments with cells, B02 was dissolved in DMSO; when injected into animals, B02 was dissolved in a vehicle containing 20% DMSO (Sigma), 20% cremophor (Sigma) and 60% NS.

### Clonogenic survival assay

To test the sensitivity of MDA-MB-231 cells to increasing doses of DNA-damaging agents in the absence or presence of B02, clonogenic survival assay was performed as described [23]. Briefly, MDA-MB-231 cells were trypsinized and reseeded in 6-well plates with a density of 500 cells/well. After overnight culture, cells were incubated 1 h in complete DMEM media containing B02 (5 µM). Doxorubicin, etoposide, topotecan and cisplatin were diluted in PBS and added to the cells when specified in indicated concentrations. The cells were exposed for 1 h, then the cells were washed by PBS three times and refreshed by the media containing B02 (5 µM). After 7–10 days, cells were fixed and stained with staining solution (0.05% crystal violet, 50% methanol in PBS); finally cell colonies were counted using an AlphaImager 3400 system with AlphaEaseFC software (Alpha Innotech Co.).

### Soft agar colony formation assay

MDA-MB-231 cells grown on 10 cm plates in complete DMEM media in the log phase were treated with B02 (5 µM) for 1 h, followed by addition of cisplatin in indicated concentrations and incubation for another 1 h. Cells were trypsinized and resuspended in complete DMEM media containing 0.3% agarose (Sigma) at 40°C; then 2 ml of the cell suspension was instantly poured over the bottom agarose (1%) layer in 6-well plates to the final density of 30,000 cells/well and allowed to solidify for 1 h at room temperature. Finally, 3 ml complete media with or without B02 (5 µM) was added over the top agarose layer. Cells were grown about 3 weeks in a CO_2_ incubator at 37°C before staining with 0.005% crystal violet for 1 h. Cell colonies were counted using an AlphaImager 3400 system with AlphaEaseFC software (Alpha Innotech).

### RAD51 foci formation *in* *vitro* and *in* *vivo*

To analyze RAD51 foci formation *in* *vitro*, 2×10^5^ log phase MDA-MB-231 cells were seeded in 3.5 cm tissue culture plates with glass coverslips and grown overnight at 37°C. Cells were then incubated with B02 in indicated concentrations for 1 h followed by 1.5 h incubation with cisplatin (32 µM). The media were then refreshed to remove cisplatin and incubation continued for another 4 h before cells were fixed with PBS containing 3.0% formaldehyde and 2.0% sucrose for 10 min and immunostained with RAD51 antibodies as described previously [23]. The fluorescence images were obtained using an Olympus IX70 inverted microscope (with 100× objective) and analyzed by iVision-Mac software (BioVision).

To measure RAD51 foci formation *in* *vivo*, tumor-burdened NCR nude mice were injected intraperitoneally (I.P.) with cisplatin (6 mg/kg), B02 (50 mg/kg), combination of both, or with the vehicle, respectively. In case of the combination treatment, B02 was injected 30 min prior to cisplatin injection. Mice were sacrificed 3 h after the treatment, small pieces of tumor tissues (∼5×5×5 mm) were dissected, washed briefly with cold PBS (8.1 mM Na_2_HPO_4_, 1.47 mM KH_2_PO_4_, 138 mM NaCl, and 2.7 mM KCl, pH 7.4) then fixed overnight in 3 ml of fresh neutral buffered formalin (10%) (Fisher Scientific) and used to prepare slides of paraffin-embedded sections of tumor tissues (0.5 mm thick) by the Pathology Diagnosis Laboratory at Drexel University College of Medicine. The tissues were deparaffinized by xylene and rehydrated stepwise by soaking subsequently 5 min in each of the following solutions: 100%, 90%, 80%, 70% ethanol and water. To retrieve the antigen the slides were boiled in 10 mM citrate buffer, pH 6.0 for 30 min. Then the slides were cooled down to room temperature and equilibrated with PBS for 5 min. The tissues were blocked by incubation in PBS containing 0.5% Tween-20, 0.1% Triton X-100 (PBSTT) supplemented with 4% (w/v) BSA and 4% donkey serum for 1.5 h at room temperature. Tissues were then incubated with rabbit polyclonal anti-RAD51 antibody (1∶500) (a gift from Dr. Golub) in PBSTT containing 4% (w/v) BSA and 4% donkey serum overnight at 4°C. The sections were washed 3 times for 5 min each in PBSTT and then incubated with 1: 100 of donkey anti-rabbit Alexa Fluor 488 antibody (Invitrogen) in PBSTT for 2 hr at room temperature. Nuclei were counterstained with DAPI in mounting media (Vector Lab.). RAD51 foci were visualized under a ZEISS LSM5 confocal microscope with 63× oil objective. The images were analyzed by Image J software, and foci were counted after subtraction of the extra-nuclei background.

### Orthotopic xenograft model

Log phase MDA-MB-231-Luc cells were collected and washed 3 times with cold PBS, pelleted down and resuspended in a cold serum-free Dulbecco's Modified Eagle Medium: Nutrient Mixture F-12 (DMEM/F-12) Media (Sigma). Then 4 parts of cell suspension were well-mixed with 1 part of pre-chilled Matrigel (BD science) and kept on ice for a shortest possible period of time before inoculation. To perform tumor cells inoculation, the female NCR nude mice, anesthetized by inhalation of ∼2% isoflurane, were injected subcutaneously with 0.5×10^6^ cells in 0.1 ml through a 27 ½ gauge needle into the fat pad located under the left side of the 4^th^ inguinal mammary gland. After 10 days, when the tumors reached 60–90 mm^3^ the mice (n = 5) were randomly distributed into treatment groups which were treated with B02, cisplation, combination of both, B02 vehicle, or NS. A control group (n = 5) was left untreated. Cisplatin and B02 were dissolved in NS and cremophor/DMSO/NS (1∶1∶3) vehicle, respectively, immediately before injection. In a combination treatment group, the mice were injected with B02 (50 mg/kg or indicated otherwise) and cisplatin (4 mg/kg or indicated otherwise). In B02 group, mice were injected with B02 and NS; in cisplatin group, mice were injected with cisplatin and B02 vehicle. Cisplatin (or NS) was administrated 3 h after B02 (or its vehicle) injection. All the treatments were executed through I.P. injections on day 11, 13, 15 and 17 after tumor cells inoculations. The volume of each individual injection was maintained constant at 100 µl. Tumor size measurements performed by a caliper and body weight measurements were carried out every other day. In caliper measurements, the tumor sizes were determined by the formula *V = a^2^b/2*, where *a* and *b* are the shortest and longest diameters of tumor, respectively [26]. Bioluminescent imaging was carried out on days 11 and 43 after tumor cells inoculation. Mice were euthanatized once the tumor size reached 10% of body weight.

### Bioluminescent imaging

The bioluminescent imaging was performed using an IVIS Lumina XR (Caliper Life Science). The images were analyzed by Living Image software (Caliper Life Science). For *in* *vitro* imaging, the MDA-MB-231-Luc cells were plated in a 6-well plate with indicated density, and then D-luciferin K (Gold Biotechnology) was added to the plate, to the final concentration 150 µg/ml. The cells were incubated 5 min before imaging. The luminescent exposure time is 1 s/plate. For *in* *vivo* imaging, the mice received I.P. injection of D-luciferin K (150 mg/kg) in PBS buffer (Cellgro). After 10–15 min, mice were anesthetized by ∼2% isoflurane through inhalation and immediately imaged with 1 s the luminescent exposure time. Tumor volumes were represented by fluorescence intensity expressed in photons/s. Background bioluminescence *in* *vivo* was approximately 9×10^7^ photons/s.

### Statistical analysis

GraphPad Prism and Excel were used for statistical calculations.

## Results

### B02 sensitizes the cancer cells to DNA damage agents

**Figure 1:**
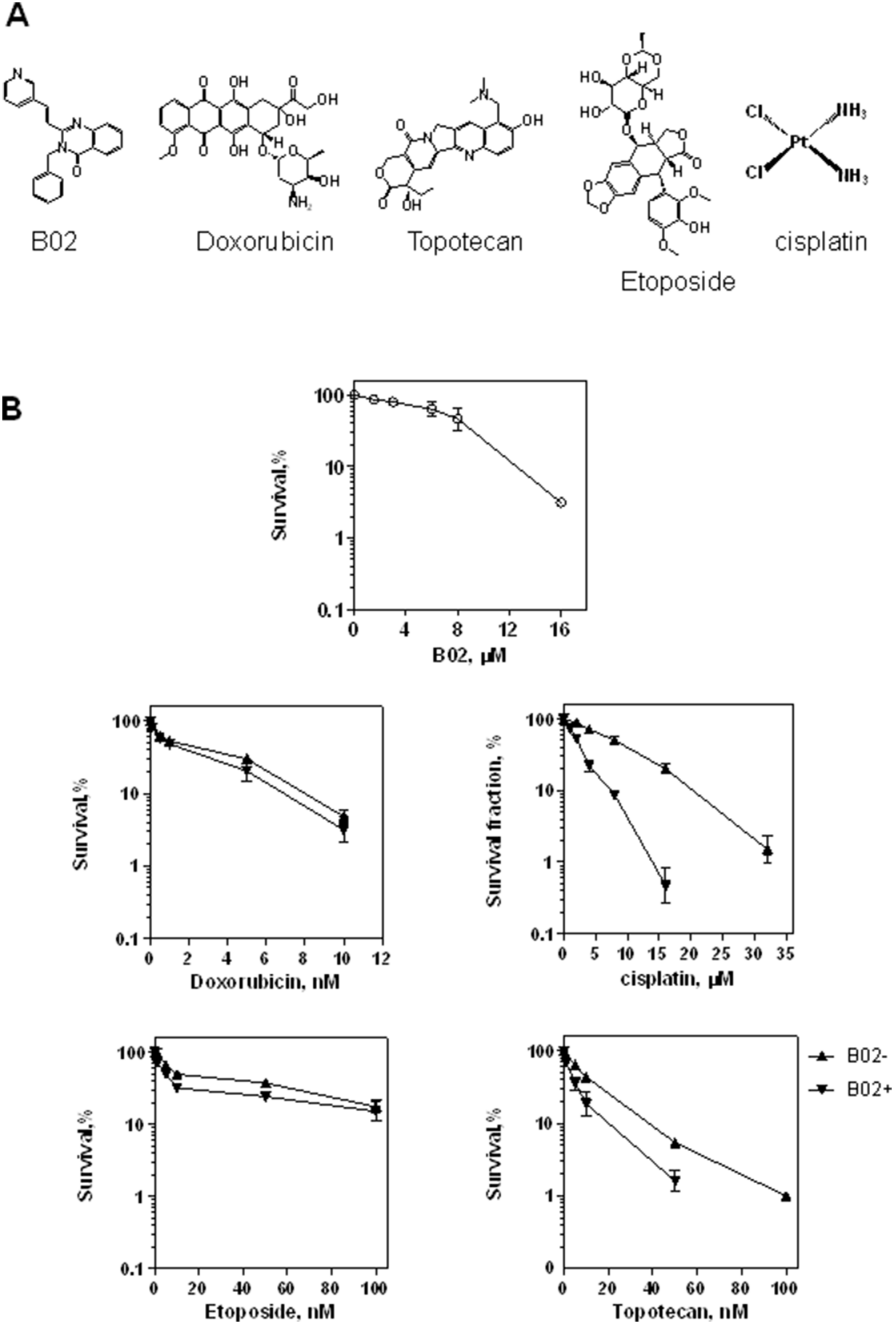
B02 increases sensitivity of MDA-MB-231 cells to DNA damaging agents. A. The structures of B02, doxorubicin, etoposide, topotecan and cisplatin. B. Survival of MDA-MB-231 cells treated with B02 (○) or with indicated agents in the absence (▴) or presence (▾) of B02 (5 µM). Experiments were repeated at least three times. Error bars represent the standard deviation (SD).

Our previous data showed that B02, a specific RAD51 small molecule inhibitor (Figure 1A), enhances the sensitivity of mouse embryonic fibroblasts (MEF) to cisplatin and Mitomycin C (MMC). However, high level of RAD51 expression in cancer cells may potentially hinder the inhibitory effect of B02. Specifically in MDA-MB-231 cells, the level of RAD51 is 8-fold and 6.6-fold higher than in their noncancerous counterparts MCF12A [27] and MCF10A [28] cells, respectively. In addition, cancer cells can excrete B02 by the P-glycoprotein pump that is generally more active in cancer cells than in normal cells [29]. Here, we tested whether B02 can enhance the sensitivity of MDA-MB-231 cancer cells to clinically approved anticancer agents, such as topoisomerase II inhibitors doxorubicin and etoposide, topoisomerase I inhibitor topotecan and DNA ICL agent cisplatin (Figure 1A). Using clonogenic survival assay we found that B02 (5 µM) increases MDA-MB-231 cells sensitivity to all these tested compounds (Figure 1B). The greatest enhancement of cancer cell killing was observed when B02 was combined with cisplatin. In the presence of B02, the IC_50_ of MDA-MB-231 cells for cisplatin decreased 4-fold. In control experiment, the toxicity of B02 was tested. The result (Figure 1B) showed that cell viability was not significantly compromised with B02 (5 µM) treatment.

### B02 enhancement of cell killing by cisplatin in the 3D-cell growing assay

**Figure 2:**
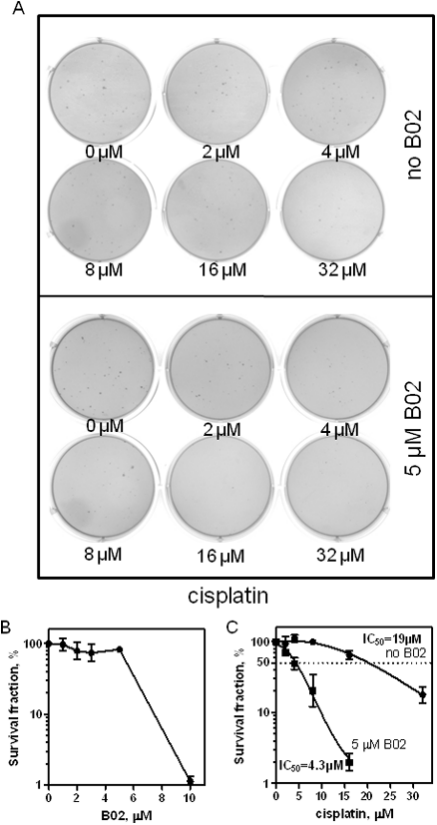
B02 increases sensitivity of 3D-growing MDA-MB-231 cells to cisplatin. A. MDA-MB-231 cells were exposed to cisplatin (in indicated concentrations) in the absence or presence of B02 (5 µM), the colonies were formed in 6-well plates coated with soft agar and stained with 0.005% crystal violet. B. The effect of B02 on survival of MDA-MB-231 cells plotted as a graph. C. The data from panel A plotted as a graph. Error bars represent SD.

Although conventional 2D cell culture is convenient for rapid evaluation of the efficacy of antitumor agents, it cannot realistically mimic the tissue morphology, microenvironment, intercellular, or cell-matrix interactions. 3D cell culture, allowing tumor cells to grow into three dimensions, turns out to be a gap-filling assay between monolayer culture and whole animal systems to test the effects of antitumor agents more accurately [30]. Therefore, we tested whether B02 sensitizes MDA-MB-231 breast cancer cells to cisplatin treatment using a soft agar colony formation assay that allows tumor cells to grow three-dimensionally in an agar matrix. In this assay, B02 significantly sensitized MDA-MB-231 cells to cisplatin treatment (Figure 2A). While alone B02 (5 µM) did not show significant toxicity to MDA-MB-231 cells (Figure 2B), it increased the IC_50_ of the cells to cisplatin approximately 4.4-fold (Figure 2C).

### B02 inhibits RAD51 foci formation induced by cisplatin in cancer cells

**Figure 3:**
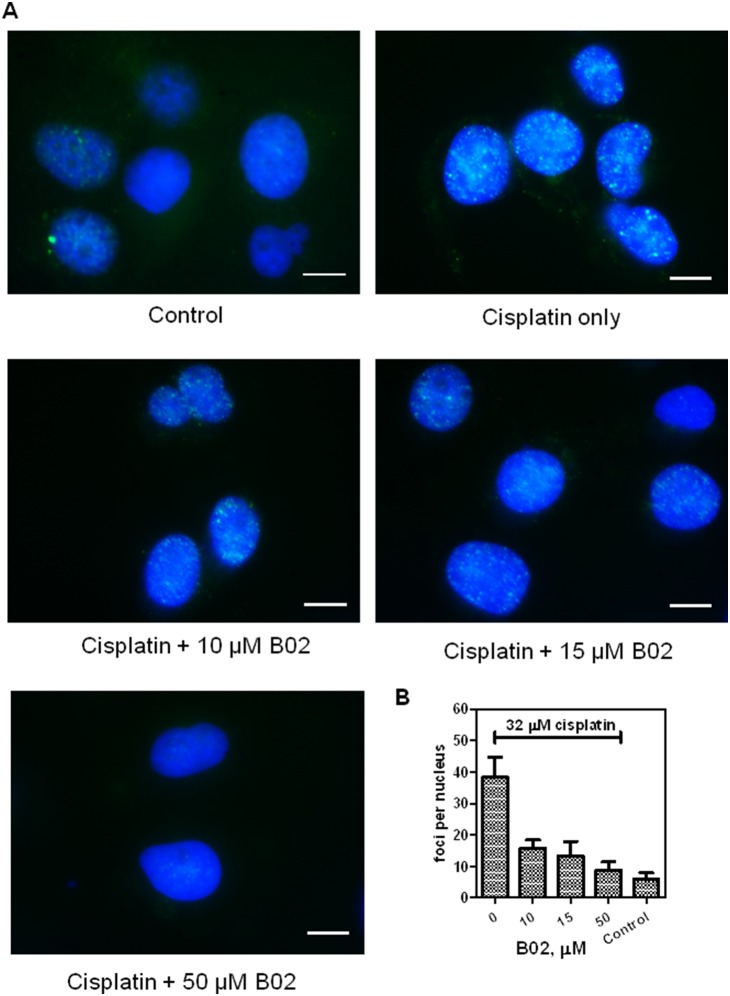
B02 inhibits RAD51 foci formation in MDA-MB-231 cells. A. MDA-MB-231 cells were treated with cisplatin (32 µM) either alone or in the presence of B02 (in indicated concentrations). RAD51 foci were visualized by immunostaining with RAD51 antibodies. Nuclei were counterstained with DAPI. RAD51 foci were visualized by an Olympus IX70 inverted microscope with a 100× oil objective. Bars indicate 20 µm. B. The mean of RAD51 foci number per nucleus was determined by counting at least 50 cells in each experiment. Experiments were repeated three times. Error bars represent SD.

We previously reported that B02 inhibited DSB-induced HR in human HEK 293 cells [23]. Here, we wanted to test the effect of B02 on HR in MDA-MB-231 breast cancer cells. First, we examined the effect of B02 on RAD51 foci formation as a manifestation of RAD51 activity in DNA repair in response to DNA damage. To induce DNA damage, MDA-MB-231 cells were treated with cisplatin (32 µM) which efficiently increased the RAD51 foci formation from 6±2 to 39±6 per nucleus (Figure 3). We found that B02 suppressed formation of RAD51 foci in response to cisplatin treatment. The effect showed a dependence on B02 concentration, as B02 in concentration 10 µM, 15 µM and 50 µM caused 70%, 79% and 91% inhibition of RAD51 foci formation, respectively. Thus, these data show that B02 has a biological effect in MDA-MB-231 breast cancer cells represented by the inhibition of DNA-damage induced RAD51 foci formation.

### B02 significantly increases the anti-tumor activity of cisplatin *in* *vivo*

We further wished to investigate whether inhibition of RAD51 would increase the anti-tumor activity of cisplatin *in* *vivo*. For this purpose, the MDA-MB-231-Luc cells were inoculated into the mammary fat pad of NCR nude mice. MDA-MB-231-luc is a stable firefly-luciferase expressing cell line derived from MDA-MB-231 cell line by neomycin selection. The cells show a high level of luminescence with luciferin intake (Figure S1). We found that the tumor signal obtained from bioluminescence imaging of mice using an IVIS Lumina XR (Caliper Life Science) showed a very good linear correlation with tumor volume determined by caliper measurement (Figure S2).

**Figure 4:**
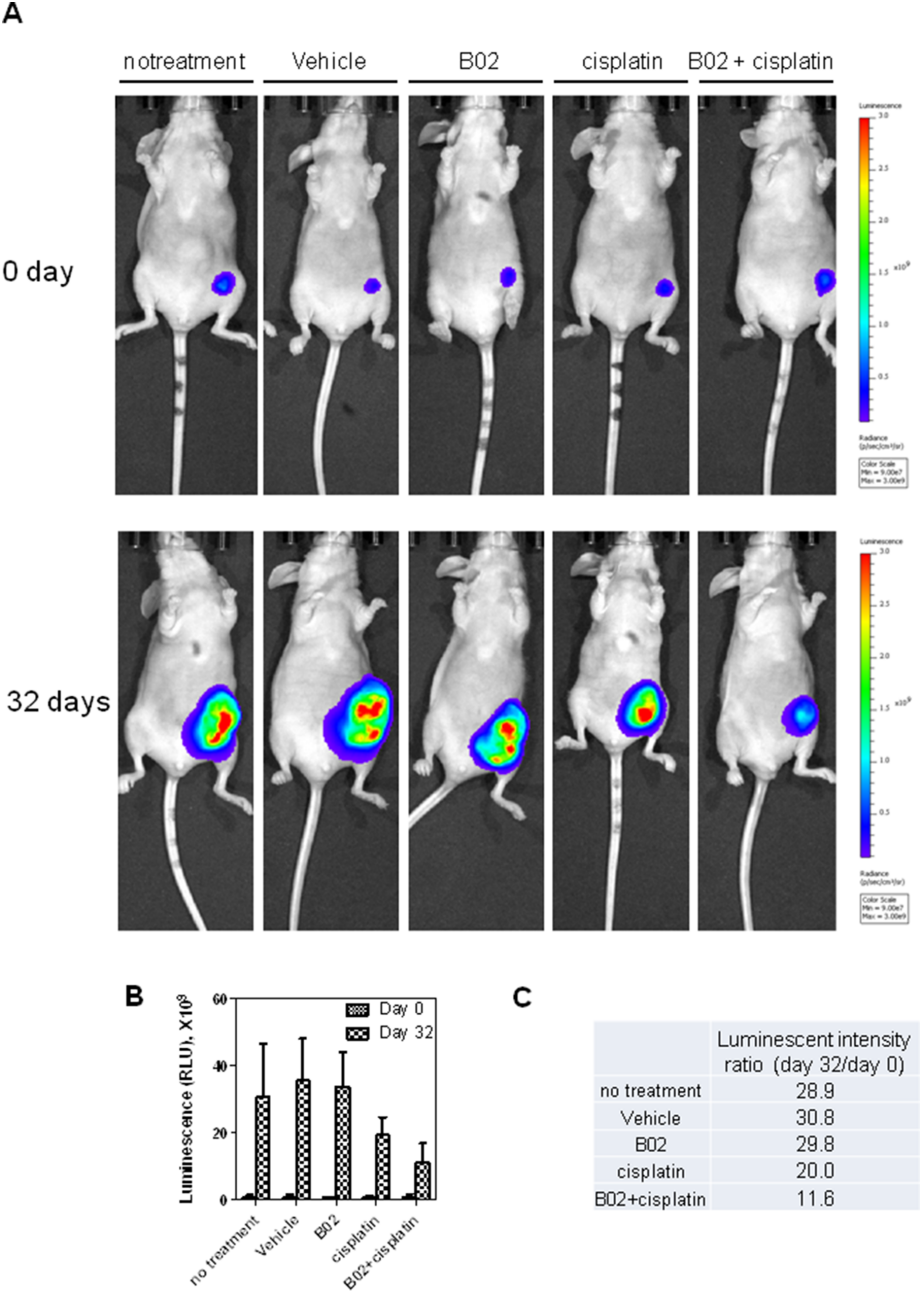
B02 in a combination with cisplatin causes a significant inhibition in tumor growth. A. The tumor images obtained 6(day 0) (top panel) and 32 days after the first treatment (day 32) (bottom panel). Mice were injected with 100 µl of D-luciferin K^+^ (GoldBio) (150 mg/kg) by I.P. and tumor cells were visualized using IVIS Lumina XR (Caliper Life Science). B. The bioluminescent intensities of tumors at day 0 and day 32 after indicated treatments were plotted as a graph. C. The ratios of bioluminescent signals observed at day 0 and day 32. Error bars represent SD.

**Figure 5:**
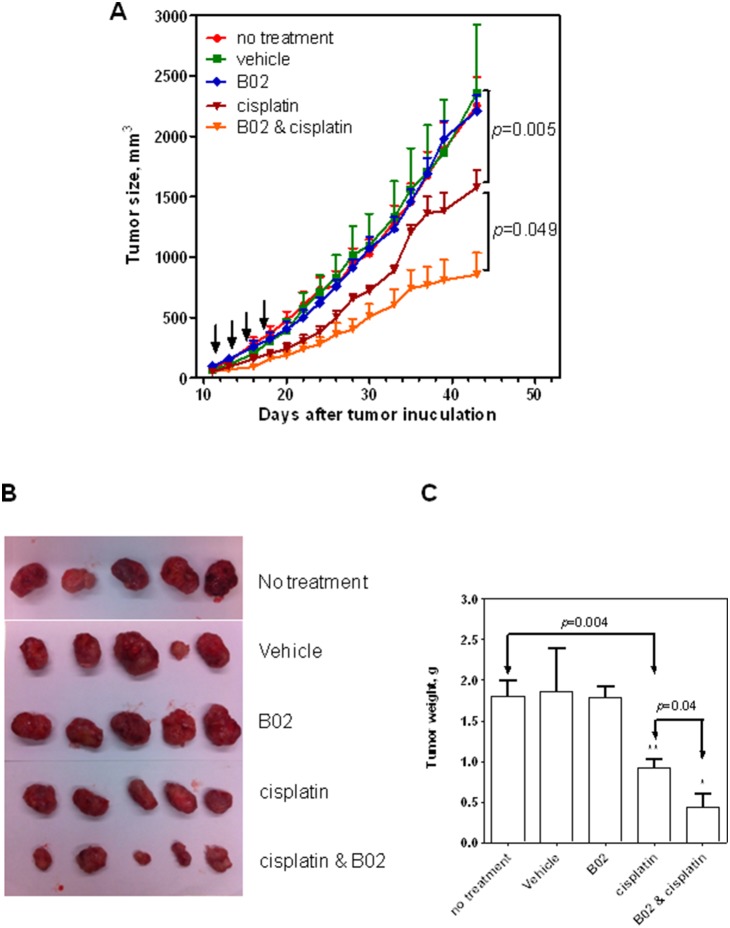
The effect of cisplatin and B02 on the time course of tumor growth, A. After 11 days of tumor inoculation, tumors were touchable. Then mice were randomly regrouped (n = 5) and treated on day 11, 13, 15, 17 with either B02 (50 mg/kg), cisplatin (4 mg/kg), or a combination of both. Mice untreated or treated with vehicle (20% DMSO, 20% cremophor, 60% NS) were shown as controls. The tumor volumes were monitored by caliper measurement in each group. The time course of tumor growth presented as a graph. Error bars represent SD. Direct measurement of the weight of tumors tissues dissected from the mice after indicated treatment, B. Mice were sacrificed 43 days after tumor inoculation and the tumors were dissected. The weight of tumors shown in panel B presented as a graph, C. Error bars represent SD.

Using bioluminescence reporter system, the tumor growth was tracked in real-time. By measuring the bioluminescent tumor signal on day 0 (the date mice were regrouped for treatment) and day 32 (the date mice were sacrificed) we found that the luminescent intensity detected from the tumors of untreated mice, mice treated with vehicle or treated with B02 (50 mg/kg, a solubility limit in vehicle) increased approximately 30-fold (Figure 4). B02 was tolerated by mice at doses up to 50 mg/kg without obvious body weight loss (Figure S3). No inhibition of tumor growth was observed on mice solely treated by B02. Mice treated with 4 mg/kg cisplatin, however, showed a 33% inhibition of tumor growth. Finally, mice treated with 50 mg/kg B02 and 4 mg/kg cisplatin showed a 66% inhibition of tumor growth. Similar results were observed in tumor growth curve measured by caliper (Figure 5A). Treatment with cisplatin caused a substantial reduction of tumor growth (*p* = 0.005). Treatment with a combination of cisplatin and B02 reduced the tumor growth further (*p*<0.05).

We also measured directly the weight of tumor tissues after mice were sacrificed (Figure 5B and C). In agreement with the data of luminescence measurement, the weight of tumors treated by a B02-cisplatin combination was significantly smaller than tumors treated with cisplatin alone (*p*<0.05).

**Figure 6:**
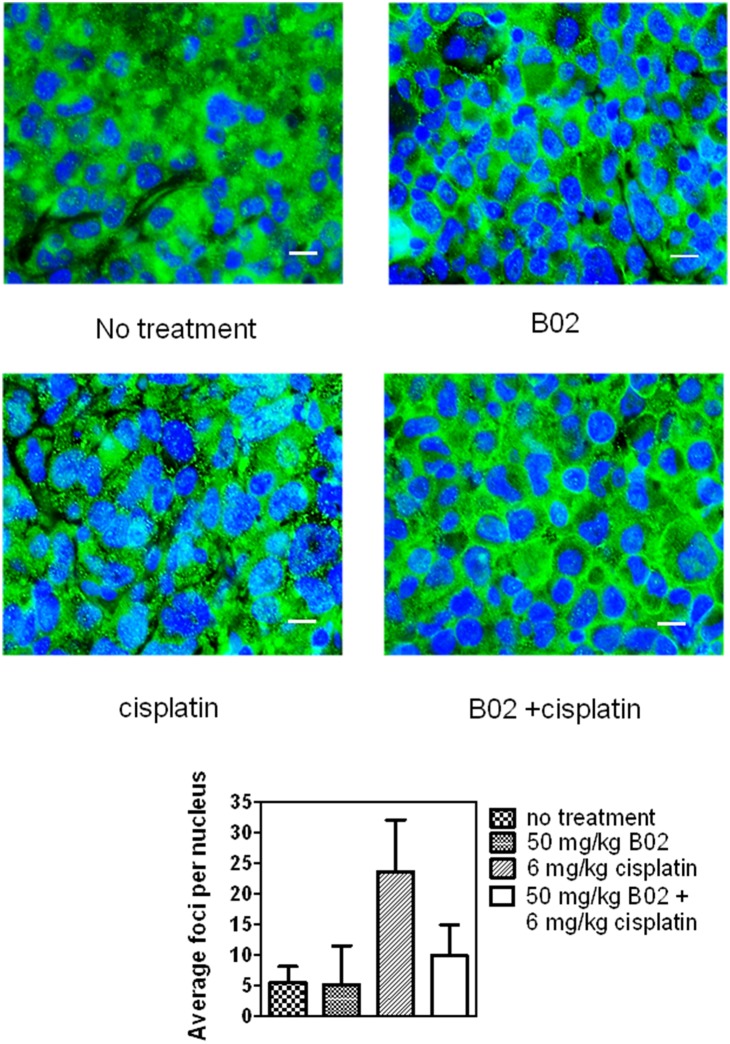
B02 inhibits the RAD51 foci formation in tumor tissue. NCR nude mice burdening with MDA-MB-231 xenograft tumors were treated by I.P. with either B02 (50 mg/kg), cisplatin (6 mg/kg), both or left untreated. The mice were sacrificed 3 h after administration, and tumor tissues were dissected and fixed in 10% formalin. Nuclei were counterstained with DAPI. After immunostaining with RAD51 antibodies, RAD51 foci were visualized using a ZEISS LSM5 confocal microscope with a 63× oil objective. Bars indicate 20 µm. The low panel shows the number of foci per nucleus in foci positive cells; it was determined by counting at least 20 nuclei in each experiment. Error bars represent SD.

To confirm that the enhancement of tumor killing *in* *vivo* by B02 is caused by targeting RAD51, we investigated the effect of B02 on RAD51 foci formation induced by cisplatin in tumor tissues in mouse xenografts. We found that treatment of tumors with B02 (50 mg/kg) significantly inhibited the RAD51 foci formation induced by cisplatin (6 mg/kg) (Figure 6; Figure S4). After subtraction of spontaneous RAD51 foci formed in the tumor tissues of the untreated mice, B02 caused a 76% decrease in the number of RAD51 foci per nucleus comparing with tumors treated with cisplatin. In a control experiment, B02 alone did not induce any RAD51 foci formation (Figure 6). Taken together, our data demonstrated that B02 significantly enhanced the tumor killing by cisplatin likely through inhibition of RAD51.

## Discussion

Approximately 25% of humans are expected to develop cancer during their lifetime and most of them will receive chemo- or radiation therapies alone or in combination with surgery. Therefore, it is very urgent to increase the efficacy of these therapies. Recently, DNA repair pathways have been recognized as an important potential target of anti-cancer therapies [8]. Due to intrinsic genome instability of cancer cells, tumorigenesis is often accompanied by mutations in DNA repair pathways [7]. In turn, mutations in DNA repair proteins may further accelerate malignant transformation by increasing mutagenesis. However, inactivation of DNA repair proteins may also expose the Achilles’s heel of tumors by making their survival dependent on the remaining alternative DNA repair pathways. Here, we wanted to exploit this cancer vulnerability by developing specific small molecule inhibitors of RAD51, a central HR protein.

HR repairs DNA damage produced by DSB- and ICL-inducing agents which are often used in chemotherapy. Because, it operates during and after DNA replication from S to G2 cell cycle phase, it is thought that HR plays an especially important role in cancer cells which remain more frequently in S/G2 phase than normal cells do [9]. HR may be considered as the last resort of DNA repair. When other DNA repair mechanisms, like base excision repair (BER) or nucleotide excision repair (NER) fail, DNA lesions are typically converted during DNA replication into DSBs. These lesions are channeled into the HR pathway, because NHEJ, another major DSB repair pathway, is generally inactive during DNA replication [16]. In addition, due to metabolic dysfunction oxidative damage increases in tumors up to 100-fold further depleting the capacity of the cellular DNA repair systems and increasing the load of HR [31], [32], [33].

Radiation therapy and genotoxic chemotherapy kill cancer cells by targeting their DNA. However, in ∼50% of cases cancer cells survive the therapy by a number of mechanisms, including upregulation of DNA repair proteins [17]. Thus, it was reported that RAD51 is almost ubiquitously overexpressed in tumors [11], [12], [13] and that RAD51 overexpression is linked with the acquired chemo- and radiation resistance of cancer cells [14], [17], [18]. Therefore, downregulation of RAD51 may not only enhance the anticancer activity of the therapeutic drugs but also slows down development of chemotherapeutic resistance. Indeed, siRNA strategies have been successfully used to enhance killing of cancer cells by cisplatin [20] and attenuate RAD51-mediated radioresistance [34], [35].

Use of small molecule inhibitors of DNA repair proteins has been recognized as an important approach in cancer therapy. Small molecule inhibitors of several DNA repair proteins have been identified and applied to clinical trials [7], [8], [36]. Previously, by high throughput screening of small molecule libraries we identified a compound that specifically inhibited DNA strand exchange activity of RAD51 [22]. We showed that B02 acts by disrupting RAD51 binding to DNA [23]. In cells, B02 inhibits RAD51 foci formation induced in human HEK293 cells by IR and increased the sensitivity of MEF to MMC and cisplatin [23]. However it remained unknown whether B02 can sensitize cancer cells in which the expression level of RAD51 is significantly increased compared with normal cells. In our current study, using a highly aggressive Triple Negative Breast Cancer cell line, MDA-MB-231, we investigated the ability of B02 to enhance the sensitivity of human cancer cells to different DNA damaging agents.

We tested whether B02 can enhance the sensitivity of cancer cells to clinically approved anti-cancer agents, such as topotecan, doxorubicin, etoposide and cisplatin. While topotecan induces DNA breaks by inhibiting DNA topoisomerase I, doxorubicin and etoposide act by inhibiting topoisomerase II. Cisplatin is DNA cross-linking agent. We found that B02 enhanced cell killing by all tested therapeutic agents, though to the different extent. The strongest effect was observed in a combination of B02 with cisplatin. In combinations with three other agents, B02 enhanced cancer cell sensitivity to topotecan stronger than to doxorubicin and etoposide. These results are consistent with the previous data on the mechanism of repair of DNA damaged by these therapeutic agents. It was demonstrated that cisplatin-DNA adducts are repaired through the HR pathway [37], [38] and cannot be repaired by NHEJ. Moreover, it was shown that cisplatin-DNA adducts inhibit DNA translocation of Ku heterodimer, an essential step of the NHEJ mechanism [39]. Similarly, it was shown that HR, but not NHEJ, dominates the repair of topotecan-induced DSBs in human immortalized fibroblasts, because inhibition of DNA-PK, one of the key NHEJ enzymes, does not sensitize cells to topotecan [40]. In contrast, DSBs induced by doxorubicin and etoposide are mainly repaired through NHEJ, although HR is also activated in response to etoposide [41].

Although studies with plate-grown cells provide important data for development of target-specific inhibitors, it is critically important to confirm the biological effect of inhibitors *in* *vivo*, because inhibitors could be inactivated in animals by enzymes such as cytochrome P-450 before they are delivered to the targets [42], or have various detrimental effects of animal health preventing their clinical applications. In the current paper, we showed that B02 potentiate the anti-cancer activity of cisplatin *in* *vivo.* Our data also demonstrate that B02 inhibits formation of RAD51 foci in tumor tissues in mouse xenografts in response to cisplatin indicating that B02 can be successfully delivered to RAD51 *in* *vivo* causing cell sensitization through targeted RAD51 inhibition.

Deployment of chemicals for *in* *vivo* investigation also raises a concern of their toxicity. It is very common that treatment by chemicals themselves compromises severely the animal health. However, no obvious toxicity of B02 to mice was observed in our *in* *vivo* test. No loss of body weight was observed with the treatment by up to 50 mg/kg of B02 (a solubility limit of B02 in the vehicle). Also, we did not find any detectable morphological changes induced by B02 in kidneys and livers, main organs for detoxification. Currently, the work is in progress to increase the potency of and consequently decrease the *in* *vivo* dosage of B02 so that it can be applied to clinical studies.

## Conclusions

Recently, several RAD51 inhibitors were reported [43], [44], [45], [46], [47]. However, while they showed inhibitory effect on the biochemical activities of RAD51 or on RAD51-dependent recombination in cell lines, their effects *in* *vivo* have not yet been tested. In the current paper, we showed that B02 potentiates the anti-cancer activity of cisplatin *in* *vivo.* Our data demonstrate that B02 inhibits formation of RAD51 foci in cancer tissues in mouse xenografts indicating that B02 acts through a targeted inhibition of RAD51 protein. Thus, our current data provide evidence that specific RAD51 inhibitors can be instrumental for improving the efficacy of chemotherapies of cancer and development of novel combination cancer therapies.

## Supporting Information

Figure S1 **The MDA-MB-231-Luc cells efficiently produce bioluminescent signals with the intensity proportional to the cell number.** Densities of both parental MDA-MB-231 and MDA-MB-231-Luc cells (in numbers indicated at the top) were incubated with 150 µg/ml luciferin K^+^ for 5 min at 37°C. Then luminescent images were visualized. B. The luminescent intensity was quantified and plotted against the cell numbers.
(TIF)

Figure S2 **Caliper and bioluminescence measurements show similar values of the tumor size.** A. The tumor volumes monitored by caliper measurement (left y axis) and by luminescent signals recorded by bioluminescent imaging (right y axis) were plotted. B. The graph showing direct correlation between caliper measurement and bioluminescent signal intensity.
(TIF)

Figure S3 **B02 treatment does not affect the body weight of mice.** Mice were injected with either vehicle or B02 (in indicated concentrations) on day 3, 5, 7 and 9. Body weights of the treated and untreated mice were monitored starting on the day of first B02 injection. The time-course of mice weight dynamics is plotted as a graph.
(TIF)

Figure S4 **B02 inhibits the RAD51 foci formation in tumor tissue.** Tumor tissues were treated, dissected, and fixed, as described in Figure Legend 7. RAD51 foci were visualized after immunostaining with RAD51 antibodies by a ZEISS LSM5 confocal microscope with a 63× oil objective. The images were analyzed by Image J software, and the extra-nuclei background was subtracted.
(TIF)
