# Supplementary figures and images for: A Small Molecule Inhibitor of Human RAD51 Potentiates Breast Cancer Cell Killing by Therapeutic Agents in Mouse Xenografts
Source: PLoS One. 2014 Jun 27;9(6):e100993. doi: 10.1371/journal.pone.0100993 (PMC4074124; doi:10.1371/journal.pone.0100993)

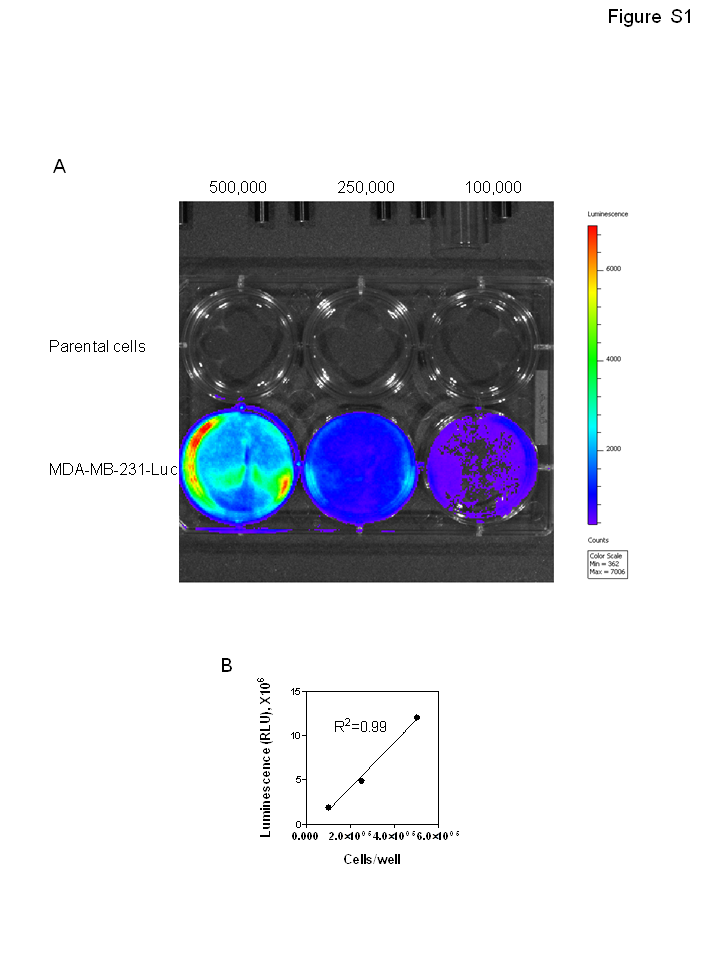

Supplement: Figure S1 — The MDA-MB-231-Luc cells efficiently produce bioluminescent signals with the intensity proportional to the cell number. Densities of both parental MDA-MB-231 and MDA-MB-231-Luc cells (in numbers indicated at the top) were incubated with 150 µg/ml luciferin K+ for 5 min at 37°C. Then luminescent images were visualized. B. The luminescent intensity was quantified and plotted against the cell numbers. (TIF) [file pone.0100993.s001.tif]

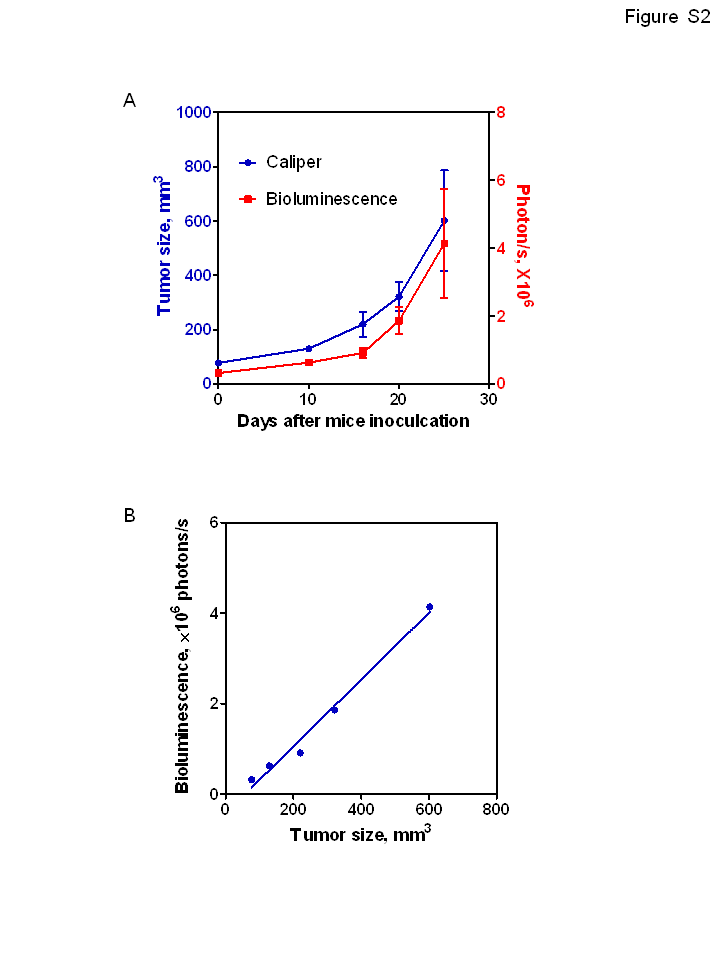

Supplement: Figure S2 — Caliper and bioluminescence measurements show similar values of the tumor size. A. The tumor volumes monitored by caliper measurement (left y axis) and by luminescent signals recorded by bioluminescent imaging (right y axis) were plotted. B. The graph showing direct correlation between caliper measurement and bioluminescent signal intensity. (TIF) [file pone.0100993.s002.tif]

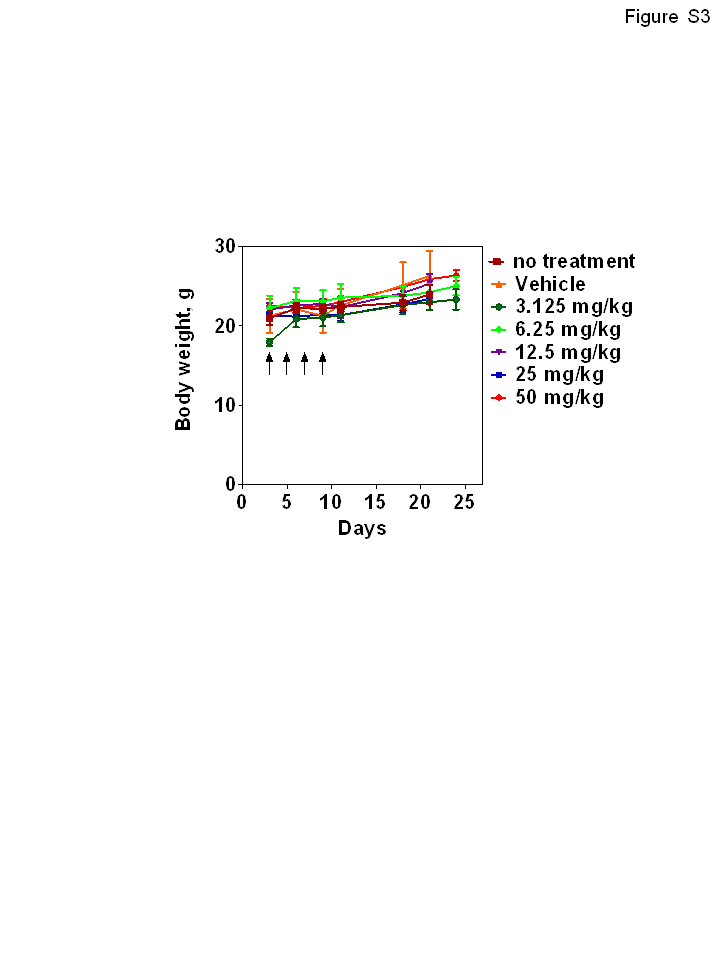

Supplement: Figure S3 — B02 treatment does not affect the body weight of mice. Mice were injected with either vehicle or B02 (in indicated concentrations) on day 3, 5, 7 and 9. Body weights of the treated and untreated mice were monitored starting on the day of first B02 injection. The time-course of mice weight dynamics is plotted as a graph. (TIF) [file pone.0100993.s003.tif]
